# DNA Methylation Regulated Nucleosome Dynamics

**DOI:** 10.1038/srep02121

**Published:** 2013-07-02

**Authors:** Isabel Jimenez-Useche, Jiaying Ke, Yuqing Tian, Daphne Shim, Steven C. Howell, Xiangyun Qiu, Chongli Yuan

**Affiliations:** 1 School of Chemical Engineering, Purdue University, West Lafayette, IN; 2 Department of Physics, George Washington University, Washington D.C

## Abstract

A strong correlation between nucleosome positioning and DNA methylation patterns has been reported in literature. However, the mechanistic model accounting for the correlation remains elusive. In this study, we evaluated the effects of specific DNA methylation patterns on modulating nucleosome conformation and stability using FRET and SAXS. CpG dinucleotide repeats at 10 bp intervals were found to play different roles in nucleosome stability dependent on their methylation states and their relative nucleosomal locations. An additional (CpG)_5_ stretch located in the nucleosomal central dyad does not alter the nucleosome conformation, but significant conformational differences were observed between the unmethylated and methylated nucleosomes. These findings suggest that the correlation between nucleosome positioning and DNA methylation patterns can arise from the variations in nucleosome stability dependent on their sequence and epigenetic content. This knowledge will help to reveal the detailed role of DNA methylation in regulating chromatin packaging and gene transcription.

DNA methylation is an important epigenetic modification that is primarily found within a CpG dinucleotide. Occurrence and removal of DNA methylation has important implications in gene regulation, e.g., X-chromosome inactivation and long-term gene silencing^1^,^2^,^3^. Multiple factors contribute to the increase in DNA methylation levels in mammals, including age, gender and environmental factors. For example, several studies have found that elder people have higher methylation levels in comparison to young adults, and males have higher global DNA methylation levels compared to females^4^. It is well-established that CpG methylation located in the promoter region plays a vital role in gene regulation. Abnormal increase in DNA methylation levels, particularly within the promoter region of tumor suppressor genes, has an established connection with various types of cancer, e.g., breast and lung cancer^5^,^6^. However, the molecular mechanism of how CpG methylation modulates gene expression remains elusive.

Recent genome-wide-association study (GWAS) and biochemical assays have revealed a very interesting correlation between nucleosome positioning and DNA methylation patterns^7^,^8^. Specifically, a 10 bp periodicity of CpG and methylated CpG (^me^CpG) dinucleotides is typically observed in the genome of eukaryotic organisms^7^,^8^,^9^,^10^,^11^,^12^,^13^. Unmethylated CpG dinucleotides predominantly exist in the minor grooves of the nucleosomal DNA facing away from the histone octamer, while methylated CpG dinucleotides (^me^CpG) dominate in the minor grooves of the nucleosomal DNA facing towards the histone octamer^7^,^8^.

This observed correlation can be potentially attributed to the change in the mechanical properties of DNA affiliated with the introduction of CpG or ^me^CpG dinucleotides, since DNA fragments have to be significantly distorted from their B-type conformation to comply with the surface curvature of the histone octamer^14^,^15^. Specifically, in eukaryotic cells, chromosomes consist of repetitive nucleosome units, i.e., a protein-DNA complex with ~145–147 bp DNA (nucleosomal DNA) wrapped around a histone octamer. Due to the helical feature of DNA, nucleosomal DNA contacts the histone octamer at 10 bp intervals^15^. These contacts are well conserved among different DNA sequences^15^,^16^. Depending on the nucleosomal location (relative position within a nucleosome), the DNA fragments are distorted to a different extent. Based on that, there are three distinctive regions within the nucleosomal DNA: 1) minor grooves facing the histone octamer (Minor Groove), 2) minor grooves facing away from the histone octamer (Major Groove) and 3) a central dyad location (Central Dyad).

DNA bases in the Major Groove display smooth bending with systematic underwinding, while DNA bases in the Minor Groove bend either in a smooth or kinked way dependent on the type of histone contacts, e.g., H3/H4 tetramer and H2A/H2B dimer^15^. The Central Dyad location refers to the pseudo-two-fold symmetry axis of the nucleosome. DNA bases located in this region are more susceptible to nuclease digestion and exhibit a less distorted conformation as compared with other nucleosomal DNA regions^17^,^18^.

This paper will elucidate how the relative position of CpG dinucleotides within a nucleosome may influence nucleosome conformation and stability, and how DNA CpG methylation alters the influence of CpG dinucleotides. To do this we specifically introduced defined CpG and ^me^CpG patterns to reconstituted nucleosomes and evaluate their effects on nucleosome conformation, as reflected in the end-to-end distance of nucleosomal DNA, and stability using Förster Resonance Energy Transfer (FRET) experiments, complemented with Small Angle X-ray Scattering (SAXS).

## Results

### Effects of defined CpG patterns on nucleosome conformation and stability

**Figure 1:**
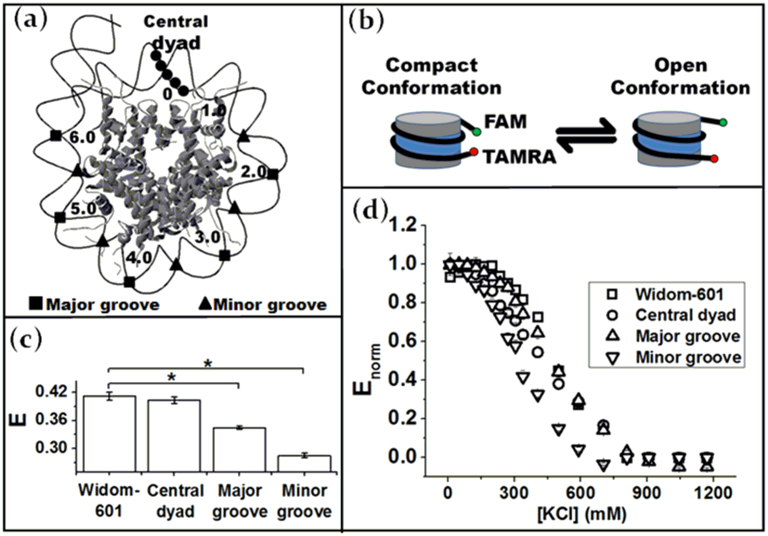
(a) Schematic drawing of the CpG patterns within a nucleosome.The numbers represent the superhelix location (SHL). (b) Schematic drawing of the DNA end breathing motion of nucleosomal DNA (c) Energy transfer efficiency of unmethylated nucleosomes at 126 mM KCl. (d) Normalized energy transfer efficiency at increasing salt concentrations. Data: mean ± standard error. *: p-value < 0.0001.

We started with the strongest-known nucleosome positioning sequence, i.e. Widom-601^12^, and then perturbed this 157 bp sequence to incorporate a specific CpG pattern as illustrated in Fig. 1(a). We introduced three specific CpG patterns in this study, namely 1) (CpG)_5_ located in the Central Dyad; 2) 5 × CpG dinucleotides at 10 bp intervals located in the Major Grooves; and 3) 5 × CpG dinucleotides at 10 bp intervals located in the Minor Grooves. The modified DNA sequence is detailed in Fig. S1. Although the Widom-601 sequence lacks any homologue sequence in nature, we chose this unique sequence, because 1) it has a well-elucidated nucleosomal DNA coordinate^16^,^19^; and 2) its large binding affinity enables us to examine specific nucleosomal locations without concerning about changes in DNA translational settings. To verify the later point, we examined all reconstituted nucleosomes using an 8% polyacrylamide gel. They all exhibited a common centralized translational setting and were free of unbound DNA (Fig. S2).

The change of nucleosome conformation, as reflected in the end-to-end distance of nucleosomal DNA, was evaluated using FRET via a time-resolved fluorescence lifetime approach. A FRET pair (Fluorescein (donor) and Tetramethylrhodamine (acceptor)) were introduced to the 5′ ends of the DNA (Fig. 1(b)) using fluorescently tagged primers (Sigma)^20^. For each DNA construct, we prepared a DNA fragment free of CpG methylation (unmethylated) and a DNA fragment with ~100% CpG methylation (Fig. S3). The FRET efficiency (*E*) measures the end-to-end distance of nucleosomal DNA and informs the compactness of the nucleosome which is related to the DNA end “breathing” motion (Fig. 1(b)) at low salt concentrations^21^,^22^. In addition, we monitored the stability of nucleosomes by collecting the energy transfer efficiencies under increasing monovalent salt concentrations (KCl ranging from 10–1200 mM). As salt concentration increases, the nucleosomal DNA will start to dissociate from a histone octamer surface^21^. Nucleosomal dynamics, such as DNA end breathing motion, H2A-H2B dimer destabilization, H2A-H2B dimer dissociation and DNA dissociation will start to contribute to the stability of mono-nucleosomes at various salt ranges^21^,^23^. Although the detailed dissociation pathway may vary among different DNA sequence constructs, we can quantify the relative stability of nucleosomes using the salt concentration at which half of the energy transfer efficiency signal is lost (*C_50_*). A similar approach was also adopted by other groups^21^,^24^,^25^.

The distinctive effects of DNA CpG patterns on the end-to-end distance of nucleosomal DNA are illustrated in Fig. 1(c) at [KCl] = 126 mM. Compared with nucleosomes with unperturbed Widom-601 sequence, additional CpG dinucleotides in either the Major or Minor Grooves can result in a more open nucleosome conformation with a larger DNA end-to-end distance. Introducing a (CpG)_5_ stretch to the central dyad region, however, does not significantly alter the nucleosome compactness. Similar phenomena have also been observed at low salt concentrations ([KCl] = 10 mM, Fig. S4). The largest conformation change was consistently observed in nucleosomes containing additional CpG dinucleotides located in the Minor Grooves facing the histone octamer.

The stability of nucleosomes, as reflected in the salt-dependent energy transfer efficiencies, exhibits dependence on DNA CpG patterns as well.(Fig. 1(d)) Nucleosomes containing the Widom-601 sequence exhibit the highest stability (*C*_50_ = 488 ± 22 mM) followed by nucleosomes with the CpG pattern in the Major Groove (*C*_50_ = 482 ± 10 mM) and the Central Dyad (*C*_50_ = 444 ± 13 mM). The dissociation curve of nucleosomes with the CpG pattern in the Minor Groove, however, was shifted significantly towards low salt concentrations (*C*_50_ = 329 ± 13 mM), suggesting reduced nucleosome stability. Further, it is worth noting that the shape of the dissociation curve of nucleosome with the (CpG)_5_ stretch in the Central Dyad is quite different from the rest. In particular, the energy transfer efficiency at low to medium salt concentrations (100–400 mM KCl) decreases with a smaller slope, suggesting a potentially different nucleosome dissociation pathway.

### Effects of defined ^me^CpG patterns on nucleosome conformation and stability

**Figure 2:**
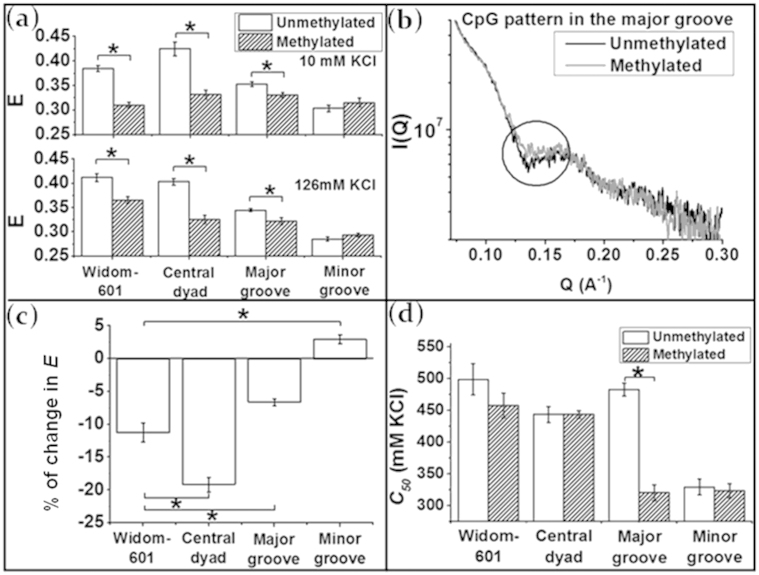
(a) Energy transfer efficiencies of all types of nucleosomes at 10 mM and 126 mM KCl.(b) SAXS profile of nucleosomes with the CpG pattern in the Major Groove at 10 mM KCl. (c) Changes in energy transfer efficiency due to the methylation of specific CpG patterns at 126 mM KCl. (d) Comparison of nucleosome stabilities with and without CpG methylation. Data = mean ± standard error. *: p-value < 0.005.

We then proceeded to quantify the effects of DNA CpG methylation on changes of nucleosome conformation and stability. Our results are summarized in Fig. 2. For all nucleosomes examined in this study, DNA methylation leads to the formation of a more open nucleosome structure with enhanced DNA end breathing motion, with the exception of nucleosomes containing additional CpG dinucleotides in the Minor Grooves which remains almost unchanged (Fig. 2(a)). The largest conformational change induced by DNA methylation were observed in DNA constructs with (^me^CpG)_5_ located in the Central Dyad.

We further examined the dependence of nucleosome conformation on DNA methylation level using SAXS. DNA end “breathing” motion has been shown in previous studies to give rise to a specific feature in SAXS profiles around the scattering vector *Q* = 0.14 Å^−1^ by smoothening the dip in the scattering curve^26^,^27^. We have thus measured the SAXS *I(Q)*s of both nucleosomes with the CpG patterns in the Major (Fig. 2(b)) and Minor Groove (Fig. S5). Fig. 2(b) shows that the methylated nucleosomes with the CpG pattern in the Major Groove have a smoother dip around *Q* = 0.14Å^−1^ (enhanced DNA end breathing motion) as compared with the unmethylated ones, consistent with the results from FRET.

To further illustrate the effects of different ^me^CpG patterns at specific nucleosomal locations, we calculated the change in the end-to-end distances of nucleosomal DNA as (*E_met,i_* − *E_unmet,i_*)/*E_unmet,i_* for each type of nucleosomes (*i*), where *E_unmet,i_* and *E_met,i_* refer to the energy transfer efficiency of a specific type of nucleosome containing 0% and ~100% DNA methylation respectively. The results are illustrated in Fig. 2(c). Interestingly, when compared with Widom-601 sequence, (^me^CpG)_5_ in the central dyad leads to a larger end-to-end distance of nucleosomal DNA (a more open conformation), while additional CpG dinucleotides in the Major or Minor Groove seem to have the opposite effect by suppressing the DNA end breathing motion and promoting a more compact nucleosome conformation compared with nucleosomes with unperturbed Widom-601 sequence. This effect is more dominant for CpG dinucleotides in the Minor Groove.

The nucleosome stability was almost unaffected by DNA methylation, with the exception of nucleosomes with the CpG pattern in the Major Groove (Fig. 2(d)). Before methylation, the stability of these nucleosomes is high and comparable to that of nucleosomes containing unperturbed Widom-601 sequence. After methylation, though, the stability of these nucleosomes drops drastically to a value comparable to that of nucleosomes with additional CpG sites in the Minor Groove.

## Discussion

The perturbations incorporated into the Widom 601 sequence to generate specific CpG patterns also affect the G + C content, the total number of CpG sites and the total number of CpG dinucleotides in each of the three distinctive nucleosomal regions, i.e., Minor Grooves, Major Grooves and Central Dyad. To identify the key parameter(s) that leads to the variations in nucleosome conformation and stability as seen in our study, we performed a Pearson's coefficient analysis^28^. This analysis identified that changes in nucleosome conformation and stability have the strongest correlation with the number of CpG sites in the Minor Groove over the other aforementioned sequence features (Table S1).

Among all four unmethylated nucleosomes examined in this study, nucleosomes containing extra CpG dinucleotides in the Minor Grooves exhibit enhanced DNA end breathing motion and reduced nucleosomal stability. These results suggest that CpG dinucleotides are not favored in the nucleosomal DNA segment whose minor groove face the histone octamer, consistent with previous GWAS results which suggest that for well-positioned nucleosomes, (T + A) tracks are preferentially located in the Minor Groove, while the (G + C) tracks are preferentially located in the Major Groove^29^,^30^,^31^,^32^,^33^. This preference is likely to originate from the ability of CpG dinucleotides to induce bending towards the major groove^34^. Consequently, when located in the Minor Groove, CpG dinucleotides render larger energetic barriers for DNA to comply with the surface curvature of the histone octamer. A more favorable curvature can be assumed for DNA constructs containing CpG sites in the Major Groove. For (CpG)_5_ stretch located in the Central Dyad, we do not expect a large curvature change. This is because although CpG dinucleotide has a high curvature, GpC dinucleotide, originated from the same CGCG repeat, has a low curvature which compensates for the effect of CpG dinucleotides^34^,^35^. Other factors, such as the change in DNA bending flexibility and stretching stiffness, are also expected to contribute simultaneously to the observed conformational and stability differences.

Comparing the dynamic conformation of nucleosomes with and without DNA methylation, our results suggest that depending on the nucleosomal locations of ^me^CpG dinucleotides, DNA methylation can have very different effects. Although, for the four DNA constructs examined in this study, DNA methylation does not further compact the nucleosome, methylation in the central dyad seems to exhibit a different trend in modulating nucleosome conformations as compared with methylation in the Major or Minor Grooves. The large conformational change induced by DNA methylation in nucleosomes with additional CpG sites in the Central Dyad can originate from the reduced DNA curvature of a typical (^me^CpG)_5_ stretch^34^,^35^. This finding is consistent with previous predictions from molecular dynamics simulations^34^. On the other hand, ^me^CpG dinucleotides at 10 bp intervals can permit more local curvature on the DNA constructs facilitating their binding to the curved surface of the histone octamer^34^.

The dramatically reduced stability of nucleosomes with additional methylated CpG sites in the Major Groove suggests that ^me^CpG dinucleotides are no longer favored in the Major Groove as previously observed for unmethylated nucleosomes. This transition can originate from the orientation of methyl side chains, since they normally stick out from DNA major grooves, compacting the minor groove and broadening the major groove^36^. Such structural features can be favorably accommodated in the minor grooves of the DNA facing the histone octamer. The preference of ^me^CpG dinucleotides to locate in the Minor Groove is also consistent with GWAS findings^7^.

The DNA methylation induced change in nucleosomal DNA end-to-end distance, as observed in this paper, does not agree with the previous report using 5S rDNA sequences^37^. The observed variation could originate from the different buffer compositions used in those studies and/or different nucleosomal locations of CpG dinucleotides. Although the findings of this paper can be a major stepping stone towards understanding the effects of DNA methylation on chromosome compactness, other factors, such as interactions between nucleosomes, effective lengths of linker DNA and interactions with linker histone proteins, have to be accounted for to correlate the observed changes in mono-nucleosome conformation with chromosome compactness. This additional information will be essential to reconcile the controversial evidences as to the effects of DNA methylation on chromosome compactness in literature^38^,^39^,^40^.

In summary, our results suggest that nucleosome conformation, as exemplified by the end-to-end distance of nucleosomal DNA, and stability are distinctively modulated by DNA CpG and ^me^CpG patterns. CpG dinucleotides at 10 bp intervals have a large effect on the compactness of nucleosomes. In particular, CpG dinucleotides located in the minor groove facing the histone octamer significantly decrease nucleosome stability and enhance DNA end breathing motion. As a result, CpG sites are less favored in the Minor Groove locations consistent with GWAS findings^7^,^8^. DNA methylation, on the other hand, differently affects nucleosome conformation depending on its location within the nucleosome. Specifically, our results based on the Widom-601 sequence suggest that methylation on the central dyad further decompacts the nucleosome, while ^me^CpG dinucleotides at 10 bp intervals seem to exhibit the opposite effect. However, for all four DNA constructs examined in this study, DNA methylation does not further compact the nucleosome, consistent with our previous findings^20^. Further, the stability of nucleosomes is significantly reduced by methylation of the CpG dinucleotides located in the Major Groove. This finding suggests that ^me^CpG sites will no longer be preferred in the Major Groove in methylated nucleosomes as seen for unmethylated nucleosomes.

The usage of Widom-601 sequence is essential in this study to accurately position the ^me^CpG dinucleotides. The current results are, thus, potentially limited to the context of the Widom-601 sequence. Although studies using other DNA sequences are essential to generalize our findings, this work is significant by elucidating a potential molecular mechanism accounting for the correlation between DNA sequence, including the fifth base of DNA, i.e., methylated cytosine, and nucleosome positioning patterns observed in eukaryotic genomes.

## Methods

### Preparation of DNA and nucleosome samples

All DNA fragments were derived from the Widom-601 sequence with detailed sequence outlined in Fig. S1. All DNA sequences were synthesized and sub-cloned into a pUC57 vector by a commercial source (GenScript, Piscataway, NJ). The accuracy of the DNA sequences was verified by DNA sequencing. For each type of DNA construct, we prepared two types of labeled DNA, i.e., a Fluorescein (FAM) labeled (donor-only labeled) and a FAM/TAMRA (fluorescein/Tetramethylrhodamine) labeled (dual-labeled) DNA, using a PCR approach as described before^20^. DNA samples, 157 bp in length produced using PCR, are free of DNA CpG methylation and exhibit almost identical electrophoretic mobility as examined using a 6% polyacrylamide gel (Fig. S6). DNA CpG methylation was introduced to all DNA constructs using a bacterial DNA methyltransferase, M.SssI (New England BioLabs, Ipswich, MA). Complete DNA CpG methylation can be achieved after incubating the DNA sample with the methyltransferase overnight^20^. The DNA CpG methylation level was verified using the digestion pattern of BstUI, whose cleavage activity is completely blocked in the presence of DNA CpG methylation as shown in Fig. S3. The methylated DNA samples were then purified using phenol-chloroform extraction followed by ethanol precipitation to remove all DNA methyltransferase and co-factors required in the DNA methylation reaction. The labeling efficiency of the unmethylated and methylated DNA samples was respectively characterized using its adsorption spectra as detailed in our previous paper^20^. The TAMRA labeling efficiency was found to be >99%, which ensures that the energy transfer efficiency as measured in this study predominately originates from the distance-dependent Förster energy transfer between the donor and the acceptor molecules.

The histone octamers used in this study were individually expressed in *E.coli* cells, refolded and purified using an established protocol^41^,^42^. The purified histone octamers were then mixed with DNA fragments containing defined sequences and DNA methylation levels at an optimized stoichiometric ratio. The mixture was then dialyzed against a series of buffers with decreasing salt concentrations. All reconstituted nucleosomes were incubated at 45°C for two hours to facilitate the positioning of histone octamers to the central location of DNA fragments. The quality of the reconstituted nucleosomes was examined in an 8% polyacrylamide gel as shown in Fig. S2. The nucleosome preparation conditions were optimized so that the final sample does not contain any free DNA.

### Time-domain fluorescence lifetime measurements

For each nucleosome sample, the FRET labels were placed on the 5′ ends of the DNA, which is 5 bp away from the DNA sites that enter/exit from a nucleosome. The location of the FRET dye is selected to 1) report the compactness of a mono-nucleosome and 2) minimize the effects of local environment of dyes, e.g., contacts with the protein surface and/or neighboring DNA fragments, which can affect the Förster distance. The anisotropy curve of both the donor-only (FAM-labeled) and the acceptor-only (TAMRA-labeled) samples were collected using steady state fluorescence. All samples exhibited anisotropy values below 0.3, suggesting that the distances calculated using the energy transfer efficiency are within 10% error^43^. All reported energy transfer efficiencies were calculated using fluorescence lifetimes measured via a time-domain fluorescence spectrophotometer (ChronosBH, ISS, Champaign, IL) similar as described in our previous study^20^,^44^. The energy transfer efficiency (*E*) is calculated following Eq. 1: 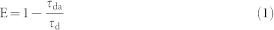
where *τ_d_* and *τ_da_* are the fluorescence lifetime of the donor-only and dual-labeled nucleosomes respectively. The concentration of all labeled nucleosome samples was kept at 1 μM by the addition of the unlabelled nucleosome samples to prevent the dissociation of nucleosomes at low concentrations^45^. As we have shown in our previous study, the donor-only sample only exhibits one fluorescence lifetime, while a dual labeled nucleosome sample exhibits two distinctive fluorescence lifetimes corresponding to the open and the closed states of a nucleosome^20^. *τ_da_* is calculated as the average lifetime following Eq.2: 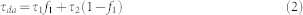
The normalized energy transfer efficiency is used to quantify the effects of salt concentrations on modulating nucleosome conformation and stability.

### Small angle x-ray scattering (SAXS) measurements

SAXS experiments were carried out at the G1 station at the Cornell High Energy Synchrotron Source in Ithaca, New York. The incident beam had an energy of 10.53 keV and a size of 250 × 250 μm. Samples of ~30 μl were injected into an in-vacuum capillary flow-cell to enable windowless data collection for background reduction. All data were collected using the same capillary with a fixed position in the beam. Radiation damage was avoided by reducing x-ray exposure time and oscillating the “plug” of the sample. Six to eight two-second exposures of the same sample were taken and no time-dependent changes were observed, indicating the absence of radiation damage. The buffer for each sample was measured before and after the sample, and the two buffer profiles were verified to be reproducible. Radial integration and correction of the raw scattering data were performed using in-house-written Matlab codes^46^, yielding the SAXS profile *I(Q)* from the nucleosome only (Fig. S5). Here *Q* = 4*π*sin(*θ*)/*λ* is the scattering vector, where 2*θ* is the scattering angle and λ is the x-ray wavelength.

## Author Contributions

I.J., J.K. and C.Y. designed the experiments, performed part of the experiments, conducted data analysis and wrote the manuscript. Y.T., D.S., S.C.H. and X.Q. performed part of the experiments, conducted data analysis and contributed to the manuscript writing. All authors reviewed the manuscript.

## Supplementary Material

Supplementary Information Suppmerntary Information
