# Supplementary material for: DNA Methylation Regulated Nucleosome Dynamics
Source: Sci Rep. 2013 Jul 2;3:2121. doi: 10.1038/srep02121 (PMC3698496; doi:10.1038/srep02121)
Supplement: Supplementary Information — Suppmerntary Information [file srep02121-s1.doc]

**DNA Methylation Regulated Nucleosome Dynamics**

Isabel Jimenez-useche^1^, Jiaying Ke^1^, Yuqing Tian^1^, Daphne Shim^1^, Steven C. Howell^2^, Xiangyun Qiu^2^ and Chongli Yuan^1*^

^1^ School of Chemical Engineering, Purdue University

^2^ Department of Physics, George Washington University

1. **Supplementary Figures**
2. **Supplementary Tables**

1. **Supplementary Figures**


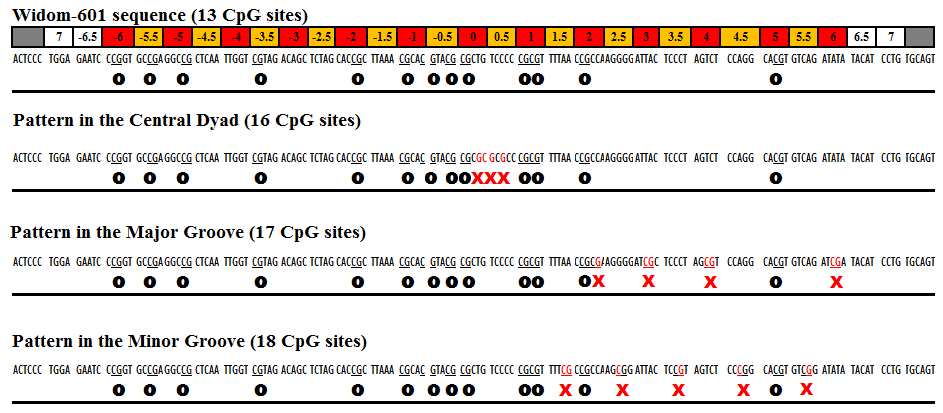


**Figure S1.** DNA sequences used in this study. The red and yellow boxes correspond to the major and minor groove locations respectively in the nucleosome. The numbers inside the boxes correspond to the superhelix locations (SHL). The black circles indicate the position of CpG dinucleotides found originally in the Widom-601 sequence and red X symbols indicate the position of the CpG dinucleotides introduced in this study. The Widom-601 sequence coordinate was adopted from Vasudevan, et.al.^6^


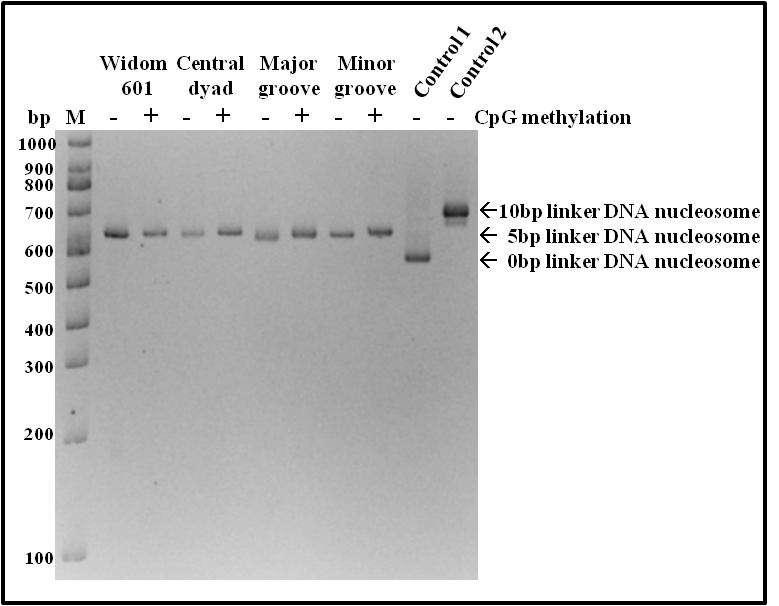


**Figure S2.** A typical 8% polyacrylamide gel of nucleosomes reconstituted with DNA fragments (157bp) containing different CpG patterns and methylation levels. Control 1 and 2: Nucleosomes with 0bp (nucleosomes with 147bp DNA) and 10bp linker (nucleosomes with 167bp DNA) DNA respectively. The gel was run at 150V, 4^o^C for 10 hours and stained with EtBr.


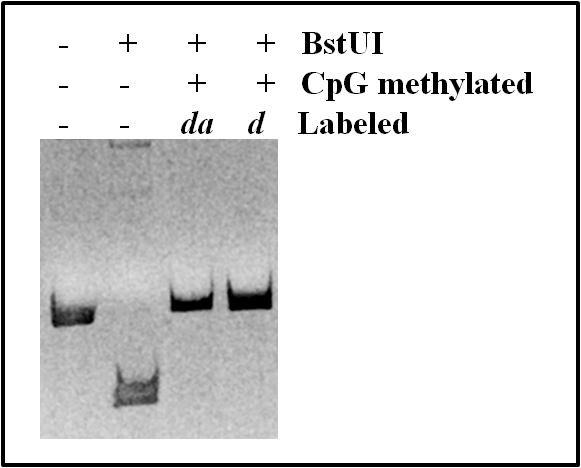


**Figure S3**. A typical BstUI digestion result of methylated and labeled DNA fragments. *d*: donor only labeled. *da*: dual labeled. The DNA is 157bp in length with additional CpG pattern in the central dyad. The gel was 6% polyacrylamide stained with EtBr


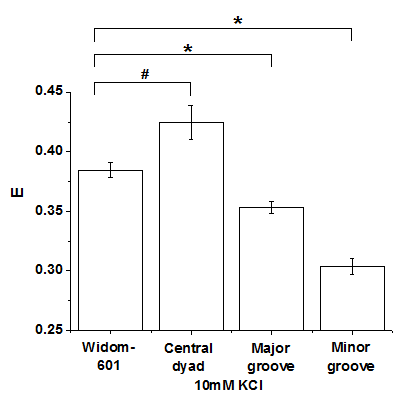


**Figure S4.** Energy transfer efficiency of unmethylated nucleosomes at 10mM KCl. Data: mean ± standard error. *: *p*-value <0.0005, #: *p*-value < 0.02.

| **(a)**  **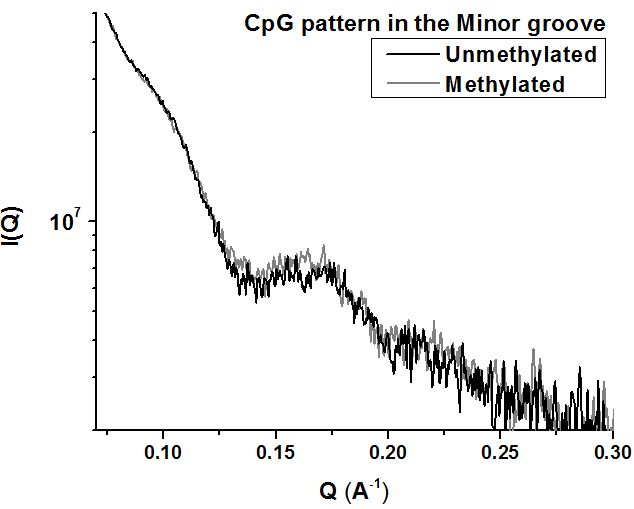** | **(b)**  **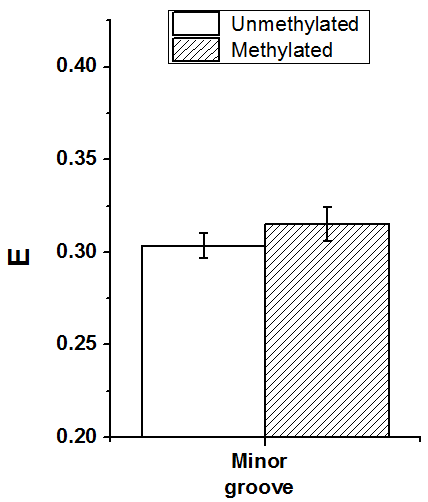** |
| --- | --- |

**Figure S5.** (a) SAXS profiles and (b) energy transfer efficiency of nucleosomes with the CpG pattern in the Minor Groove. The difference in energy transfer efficiency between unmethylated and methylated nucleosomes is not statistically significant, with a *p*-value of 0.311.


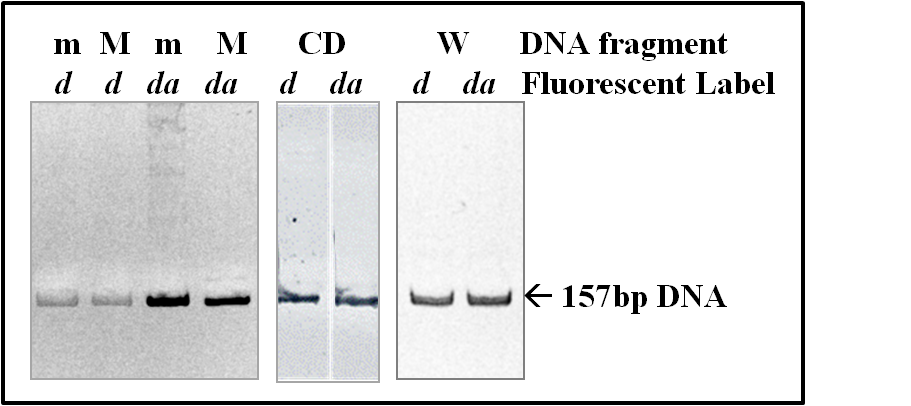


**Figure S6.** Labeled DNA fragments produced by PCR. All DNA fragments are 157bp long. *da*: dual-labeled DNA (FAM/TAMRA). *d*: donor-only labeled DNA (FAM). The DNA fragments are the Widom-601 DNA sequence (W), the DNA fragment with the CpG pattern in the Central Dyad (CD), in the Major Grooves (M) or in the Minor Grooves (m).

1. **Supplementary Tables**

**Table S1.** Pearson’s coefficient of the conformation and stability of the nucleosomes with the DNA sequence features.

|  | ***Total CpG sites*** | ***CpGs in major groove*** | ***CpGs in minor groove*** | ***CpG at Central dyad ± 5bp*** | ***%***  ***G+C*** |
| --- | --- | --- | --- | --- | --- |
| ***Conformation*** | 0.83 | 0.16 | **0.88** | -0.47 | 0.74 |
| ***Stability*** | -0.69 | 0.42 | **-0.95** | 0.03 | -0.36 |
